# Workplace, Household, and Personal Predictors of Pesticide Exposure for Farmworkers

**DOI:** 10.1289/ehp.8529

**Published:** 2006-02-16

**Authors:** Sara A. Quandt, María A. Hernández-Valero, Joseph G. Grzywacz, Joseph D. Hovey, Melissa Gonzales, Thomas A. Arcury

**Affiliations:** 1 Wake Forest University School of Medicine, Winston-Salem, North Carolina, USA; 2 University of Texas, M.D. Anderson Cancer Center, Houston, Texas, USA; 3 University of Toledo, Toledo, Ohio, USA; 4 University of New Mexico, Albuquerque, New Mexico, USA

**Keywords:** agricultural workers, folk belief, personal protective equipment, psychosocial stressors, safety behavior

## Abstract

In this article we identify factors potentially associated with pesticide exposure among farmworkers, grade the evidence in the peer-reviewed literature for such associations, and propose a minimum set of measures necessary to understand farmworker risk for pesticide exposure. Data sources we reviewed included Medline, Science Citation Index, Social Science Citation Index, PsycINFO, and AGRI-COLA databases. Data extraction was restricted to those articles that reported primary data collection and analysis published in 1990 or later. We read and summarized evidence for pesticide exposure associations. For data synthesis, articles were graded by type of evidence for association of risk factor with pesticide exposure as follows: 1 = association demonstrated in farmworkers; 2 = association demonstrated in nonfarmworker sample; 3 = plausible association proposed for farmworkers; or 4 = association plausible but not published for farmworkers. Of more than 80 studies we identified, only a third used environmental or biomarker evidence to document farmworker exposure to pesticides. Summaries of articles were compiled by level of evidence and presented in tabular form. A minimum list of data to be collected in farmworker pesticide studies was derived from these evidence tables. Despite ongoing concern about pesticide exposure of farmworkers and their families, relatively few studies have tried to test directly the association of behavioral and environmental factors with pesticide exposure in this population. Future studies should attempt to use similar behavioral, environmental, and psychosocial measures to build a body of evidence with which to better understand the risk factors for pesticide exposure among farmworkers.

Human exposure to the pesticides that exist in the home, workplace, and community is regulated by a variety of behaviors and environmental factors. While many of these factors are commonly accepted in research on farmworker health and form the basis of pesticide safety education, there has been no comprehensive review of the empirical evidence linking these factors to exposure or to the relationship of exposure and health. We focus on the measurement of behavioral and environmental factors important at the following two points in the pesticide and health relationship: *a*) those that predict pesticide exposure, including who is exposed and how he or she is exposed, and *b*) those that modify the absorbed dose of pesticides.

We based this review on the premise that such a compilation of data will allow scientists to identify factors that have been found to be associated with pesticide exposure and, perhaps more importantly, to identify the gaps in current knowledge of the pesticide and health relationship. To the extent that determinants of exposure can be assessed with comparable measures across studies, results of such studies can then be compared to provide better-grounded answers to questions on the health effects of pesticides.

In this article we present a model of the relationship between predictors of pesticide exposure among farmworkers and pesticide exposure on health outcomes. We identify comprehensively the range of factors that may be associated with pesticide exposure, and we distinguish those for which a firm relationship with farmworker exposure has been identified in the scientific literature and those for which the association can only be inferred from other data. We also suggest a minimum set of measures that are necessary to understand farmworker pesticide exposure.

## Conceptual Model

**Figure 1:**
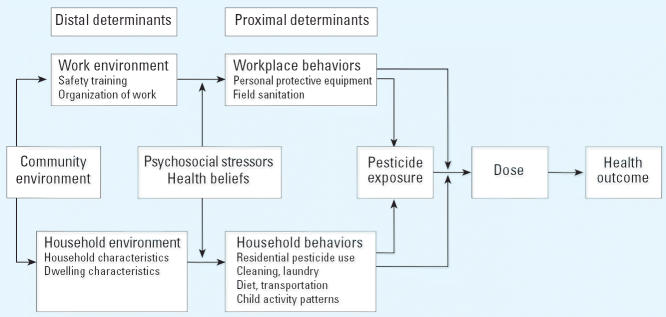
Conceptual model of the relationship between the predictors of pesticide exposure among farmworkers and their relationship to health outcome.

This article is guided by a model (Figure 1) that contrasts the proximal and the distal determinants of pesticide exposure. Those determinants that are proximal to pesticide exposure—that is, the immediate determinants of exposure—are generally behaviors practiced either by farmworkers in the workplace or by farmworkers or their co-resident household members at home. These determinants include (in the workplace) use of personal protective equipment (PPE) and field sanitation, as well as (at home) laundry practices and child activity patterns. These proximal factors are themselves determined by predictors that are considered more distal to the exposure. These predictors include environmental conditions at work (e.g., safety training), at home (e.g., number of farmworkers in residence), and in the larger community (e.g., total farmland treated with pesticides). These environmental factors affect exposure through behavior; the association of environmental and behavioral factors is moderated by psychosocial factors, including the attitudes, values, beliefs, and knowledge held by farmworkers. For example, farmworker residences with a high residential density might be expected to store soiled work clothing that would present an exposure risk to household residents. This relationship could be positively influenced by beliefs that pesticides are harmless, or negatively influenced by knowledge of recommended laundry practices.

A portion of pesticides to which an individual is exposed is absorbed as the pesticide dose, and this dose can have health effects. According to the model, the amount absorbed is moderated by some of the workplace and household behaviors (e.g., hand washing by workers or household residents) as well as by other factors. The latter moderators include genetic factors, body size, and developmental status; these characteristics are not covered in this review.

## Methods

This review focuses on the conceptual model (Figure 1) developed by the authors. Components of the model were expanded to produce a list of factors potentially related to pesticide exposure in farmworkers. These factors formed the search terms for our review of the literature that searched the PubMed, (http://www.ncbi.nlm.nih.gov/entrez/query.fcgi?DB=pubmed); Science Citation Index and Social Science Citation Index (http://portal.isiknowledge.com/portal.cgi/wos?Init=Yes&SI D=D112jMPBmi56JK4eA1); PsycINFO (http://www.psycinfo.org/psychoinfo/); and AGRICOLA (http://agricola.nal.usda.gov/) databases. We restricted reviews to peer-reviewed publications from studies that involved primary data collection and that were published in 1990 or later. A few earlier studies were included for topics with little research coverage. Articles were graded by the type of evidence for the association of a particular risk factor with pesticide exposure, as follows: 1 = association demonstrated in farmworkers; 2 = association demonstrated in nonfarmworker sample; 3 = plausible association proposed for farmworkers; or 4 = association plausible but not published for farmworkers. To be classified as “1,” the study participants had to be described as migrant or seasonal farmworkers. In most other cases the study participants were described as “growers,” “farmers,” or members of their families, and they were classified as nonfarmworkers. Study participants described as “applicators” were classified as nonfarmworkers. Summaries of articles were compiled by level of evidence and presented in tabular form. Because of space restrictions, only those articles graded “1” or “2” are presented here (Table 1). A minimum list of data to be collected in farmworker pesticide studies was derived from these evidence tables (Table 2).

## Workplace Behaviors

Wearing PPE is one of the behaviors most widely assumed to protect workers from pesticide exposure. The label PPE can apply to everything from long-sleeve shirts to protective coveralls and respirators. Studies in the United States and abroad show that wearing PPE appropriate to the task results in lower exposure to pesticides (Table 1). Although the studies vary with regard to the types of chemicals investigated, the PPE tested (gloves, overalls), and the types of exposure measured [cholinesterase activity, skin wipes, organochlorine pesticide (OCP) serum levels], they all indicate that PPE is effective in reducing worker exposure to pesticides (Fenske et al. 1990; Gomes et al. 1999; Hernández-Valero et al. 2001; Lander et al. 1991; Ohayo-Mitoko et al. 1999). Studies in farmers (Arbuckle et al. 2002) and applicators (Fenske et al. 2002a; Nigg et al. 1993) lend further support to the effectiveness of PPE, although they also indicate variations because of fabrics and clothing design. In general, fabric less capable of penetration and designs that cover the largest amount of skin provide the greatest protection from pesticide exposure for workers. Despite the indications of efficacy, studies (particularly of farmers and applicators) show that PPE is frequently not used (e.g., Perry et al. 2002).

Other worker behaviors have been suggested as ways to reduce pesticide exposure, and these alternatives are included as recommended practices in the U.S. Environmental Protection Agency Worker Protection Standard (WPS) training (U.S. Environmental Protection Agency 1992). These behaviors include washing hands in the field before eating and after mixing pesticides. The importance of such behavior is demonstrated by studies showing that pesticides can be transferred to the home via automobile (e.g., Curl et al. 2002; Thompson et al. 2003). Curwin et al. (2003) showed that farmworker hand levels of the OP acephate could be reduced 96% by handwashing.

Additional practices have been suggested to reduce exposure. These practices include wearing grower-provided uniforms and showering at the worksite before returning home. There have been no tests to determine if such workplace behaviors would reduce exposure of the farmworker or the farmworker family.

Farmworker children are sometimes taken to the fields either to work or because adequate child care is lacking (Cooper et al. 2001). Such practices are likely to be predictors of pesticide exposure. Hernández-Valero et al. (2003) investigated the possible pathways of OCP exposure among 36 migrant farmworker children whose home base was Baytown, Texas. One-third of the children had previously conducted farmwork, and the farmwork duration significantly increased their exposure levels. Mandel et al. (2005) found that children of Minnesota growers often helped apply chemicals and, therefore, had levels of pesticide exposure closer to those of the parent who applied chemicals than to the other parent.

## Household Behaviors

The application of residential pesticides in the home and yard has been investigated as a source of pesticide exposure among farmworkers and nonfarmworkers (Table 1). The collection of wipe (Quandt et al. 2004) or vacuum samples (Bradman et al. 1997), which allow direct identification of the type of pesticide found, has been used to link pesticides applied to worker dwellings to those pesticides detected. However, not all studies have had positive results (McCauley et al. 2001). Urinary metabolites of OP pesticides have also supported the link between residential pesticide application and worker exposure (Arcury et al. 2005).

Similar results have been found in nonfarmworker populations. Yard and garden pesticides were found to be transferred into homes by residents and by dogs (Lewis et al. 2001, Morgan et al. 2001; Nishioka et al. 2001). Use of OP pesticides in gardens is associated with metabolite levels in children (Fenske 2002b; Lu et al. 2001).

Several household sanitation behaviors are associated with farmworker pesticide exposure. Bradman et al. (1997) found that more frequent mopping and vacuuming was associated with lower pesticide recoveries in dust wipes. Arcury et al. (2005) suggested that having a vacuum cleaner was associated with lower levels of urinary OP metabolites.

A number of studies have documented the high potential for personal exposure to pesticides caused by waiting for extended periods before showering after work, not changing clothes immediately after work, and failure to separate work from household laundry (Alavanja et al. 1999; Curwin et al. 2002; Goldman et al. 2004). However, with the exception of McCauley et al. (2003), there is little direct evidence to support this association.

## Work Environment

The organization of work is a subfield of occupational health that is concerned with the way that work processes are structured and managed. Organization of work investigators attend to such factors as the nature of the employment relationship (e.g., permanent versus contingent labor), job design (e.g., complexity of tasks and level of worker control), interpersonal elements of jobs (e.g., worker–supervisor relations), as well as such things as work schedules, job security, and communication with an employing organization. Although it has not been explicitly used in farmworker research, evidence suggests that several aspects of the way farm work is organized contribute to pesticide exposure (Marquart et al. 2003).

Several interrelated processes underlying the nature of the employment relationship suggest that pesticide exposure is likely to be greater among farmworkers in seasonal (e.g., workers with H2A visas) or day labor relationships in contrast to those in more “permanent” positions. Farmworkers in employment relationships that are more permanent may receive more effective safety training and more consistent reinforcement of safety behaviors than seasonal farmworkers or day-laborers. Researchers contend that workers in nonstandard employment relationships, such as seasonal workers or day-laborers, may be given tasks that place them at greater risk of becoming exposed to pesticides compared to permanent workers (Quinlan et al. 2001). Moreover, farmworkers in seasonal and day-labor arrangements may be less likely to request safety equipment or to report potential hazards to owners/operators out of fear that it may jeopardize future opportunities for work (Aronsson 1999; Aronsson et al. 2002; Quinlan et al. 2001). Despite the plausibility of several of these linkages, differences in pesticide exposure among farmworkers in different types of employment relationships have not been studied explicitly.

Different aspects of job design, or the tasks performed on a job and how they are performed, have been linked to pesticide exposure (Table 1). Tasks that are not regulated by the WPS can result in elevated pesticide exposure (Coronado et al. 2004). A great number of tasks or duties that put individuals in contact with pesticides or pesticide residues, such as self-service and repair of application equipment among applicators and a greater number of field activities among workers, are associated with more exposure (Alavanja et al. 1999; Hernández-Valero et al. 2001). Environments that provide farmworkers with little control over how pesticides are applied (e.g., high-exposure application methods), when pesticides are applied (e.g., avoiding windy days), and frequency of application are all associated with increased pesticide exposure among farmworkers (Mage et al. 2000; Martin et al. 2002; Mekonnen and Agonafir 2002). Similarly, environments that provide little personal control over protective behaviors, such as absence of well-maintained PPE or inability to wash or change clothes during the workday, contribute to elevated pesticide exposure (Alavanja et al. 1999; Arcury et al. 2002; Austin et al. 2001; Mekonnen and Agonafir 2002; Parrott et al. 1999).

Although there have been no explicit comparison studies, it is likely that different crops are associated with different levels of pesticide exposure because of the differences in tasks associated with crops. For example, some will involve greater hand labor for cultivation and harvest than others. It is likely that those requiring more hand labor will result in greater exposure.

Interpersonal elements of farm work also contribute to pesticide exposure. Better-quality relationships between workers and farmers/growers are important for identifying potential sources of pesticide exposure as well as for designing and implementing effective strategies for minimizing exposure (Grieshop et al. 1996). Communication difficulties caused by language differences between workers and farmers/growers contribute to greater pesticide exposure through less effective training (McCauley et al. 2002; Rao et al. 2004). Similarly, differences in belief systems about the risks of pesticide exposure and appropriate behaviors for minimizing risk can contribute to elevated exposure by undermining the effectiveness of training and safety programs (Arcury et al. 2001; Quandt et al. 1998; Rao et al. 2004). The psychological demands of the work environment can also contribute to lower adherence to safety regulations (Kidd et al. 1996; Thu 1998; Walter et al. 2002). Despite the strong suggested connection of these work environmental factors to pesticides, no studies have examined pesticide exposure and the organization of work, either in farmworkers or in other populations.

One of the major aspects of the work environment directly related to pesticide exposure is safety training for workers. Minimum content and standards for pesticide safety training are part of the WPS, which mandates training for field workers as well as for applicators. A number of studies have examined safety training in farmworkers, but none of these have examined the association of safety training with pesticide exposure. This work shows that many farmworkers fail to receive training as mandated (Arcury et al. 1999; Elmore and Arcury 2001; U.S. General Accounting Office 2000) but that the rates vary over time (Arcury et al. 2001). Salazar et al. (2004) found that even when safety training is presented, it is sometimes understood poorly because of language barriers. Research with applicators (Martinez et al. 2004) and farmers (Perry and Layde 2003) shows that safety training produces increased knowledge, but it does not necessarily result in appropriate safety behaviors.

## Household Environment: Physical and Social

Proximity of dwellings to agricultural fields treated with pesticides has been suggested as a dwelling characteristic associated with exposure (Fenske et al. 2000). Studies of dust samples from farmworker residences support this suggestion, both in terms of concentrations of pesticides (McCauley et al. 2001) and in numbers of pesticides found in the home (Quandt et al. 2002, 2004). Curl et al. (2003) found no association between distance to field and levels of metabolites found in children’s urine. However, these metabolite levels were associated with house dust concentrations, which, in turn, were associated with the dust in cars of farmworkers, thereby indicating a pathway from worksite to home. Among nonfarmworkers, distance from dwelling to fields was associated with concentrations in house dust (Fenske et al. 2002b; Lu et al. 2000). This linkage was reflected in higher urine concentrations of metabolites in some (Loewenherz et al. 1997) but not all (Fenske et al. 2002b) studies measuring urinary metabolites.

Various housing quality indicators have been linked to greater pesticide exposure for farmworker families. Older dwelling age (Bradman et al. 1997) and renting rather than owning (Arcury et al. 2005) have been examined. These studies were based on the belief that the greater age of a house as well as a history of different tenants might lead to the accumulation of larger amounts of pesticides, both simply as a matter of time and because there might be more opportunity for pest infestations to which pesticides are applied. Both of these measures have been linked to exposure. Quandt et al. (2004) used an interviewer’s judgment of how difficult or easy a house was to clean, reasoning that houses more difficult to clean would have a less thorough elimination of pesticides. Cleaning difficulty was associated with greater pesticide exposure.

Several aspects of the household social environment related to household composition have been suggested as major influences on pesticide exposure at home. The logic is that more persons in the household, particularly more farmworkers, will increase the volume of take-home pesticides, and this situation might be most extreme in cases of crowding. The simplest measure, total household size, has been linked to pesticides in two studies of farmworkers (Arcury et al. 2005; McCauley et al. 2001). These findings are supported by the study of Goldman et al. (2004) of pesticide-related behaviors. They found that larger household size was associated with fewer in-home safety behaviors. McCauley et al. (2003), in a study of nonfarmworker agricultural households, found weak and nonsignificant associations between household size and OP residues. More specific measures of household social environment (number of adults and number of agricultural workers in the household) have been suggested. However, this association generally has been tested by comparing agricultural and nonagricultural households (Bradman et al. 1997; Lu et al. 2000; Simcox et al. 1995), not by looking at the variation in number of adults within farmworker homes. Exceptions are the work of Arcury et al. (2005) and Quandt et al. (2004), which compared nuclear family households with those that comprised other adult relatives or nonrelatives and appeared to find more pesticides in the latter. This finding may be caused by greater track-in of pesticides with more adults, or by culture-specific issues. The investigators found that women residing in farmworker homes reported difficulty in enforcing standards of household cleanliness when male in-laws lived with the family because gender roles limit the authority of women over the behavior of fathers-in-law and other relatives. Only two studies have used density or crowding (e.g., persons/room and persons/square foot) as measures of the household social environment. McCauley et al. (2001) found no association in homes of farmworkers, and only a slight association in homes of other agricultural workers (McCauley et al. 2003).

## Community Environment

Several different measures have been used to associate overall use of pesticides in a community with exposure. None has focused specifically on farmworkers. Fenske et al. (2000) found that a majority of children in an agricultural region from both agricultural and nonagricultural families had urinary metabolites for OPs. Similar results were reported by Koch et al. (2002), who found no differences because of parental occupation or residential proximity to fields. Lee et al. (2002) measured airborne agricultural pesticides at monitoring stations in California communities. They found that the level of exposure exceeded reference values for noncancer health effects for half of the population.

In agricultural communities, historical use of some persistent pesticides may have led to long-term contamination of the soil. In areas where lead arsenate was used extensively, soil samples have demonstrated the persistence of arsenic (Wolz et al. 2003). DDT, an OCP, is still found in soil samples despite its having been removed from use decades ago (Miersma et al. 2003).

## Factors Moderating Behavior and Environment

### Psychosocial stressors

Two pathways have been proposed by which psychosocial stressors might lead to pesticide exposure of farmworkers or of growers (Figure 1). None of the studies of these stressors have actually measured pesticides, so no data have been gathered with which to validate these pathways. The first pathway is through stressors on the farmworkers, primarily the result of their social position as immigrants and the process of acculturation that they undergo. Vega et al. (1985) found that Mexican American farmworkers experience high levels of psychiatric symptoms. These symptoms are associated with limited social mobility, transience, poverty, discrimination, and a high rate of traumatic life events. These findings were supported by Hovey et al. (2002a, 2002b), who found that farmworkers suffer from high rates of anxiety. This anxiety, in turn, is associated with elevated acculturative stress, low self-esteem, ineffective social support, and lack of control over the migrant lifestyle. Looking specifically at female farmworkers, Carruth and Logan (2002) documented high levels of depressive symptoms, which were predicted by poor health, perceived hazards of farm work, having experienced recent farmwork-related injuries, and engaging in farm work over long periods of time. These documented stressors and associated mental health deficits may lead farmworkers to take more risks and to neglect to practice safety behaviors protective against pesticide exposure.

The second pathway is through stressors on growers and workers that result from the organization of farm work. Thu (1998) proposed that the narrow temporal window for growing and harvesting, long work hours in isolated work conditions, and the psychological stress associated with farming can push farmers to minimize safety standards. Others have argued that the psychological and physical demands of the job confronted by day-laborers, including farmworkers, directly promote accidents and injuries through fatigue and distraction (Kidd et al. 1996; Salazar et al. 2004; Thu 1998; Walter et al. 2002). They also argue that other difficulties faced by farmworkers, including economic hardship and job insecurity, further elevate the risk of exposure and exacerbate health effects of exposure because farmworkers who have few other employment options may fear requesting PPE or may work through dangerous situations.

### Pesticide knowledge and beliefs

Farmworkers’ knowledge about pesticides has generally been measured relative to prevailing scientific data, while beliefs come from more exploratory, ethnographic investigations. However, conceptually, both provide workers with information upon which they base their actions, so the distinction is somewhat artificial. Farmworker beliefs and knowledge have been collected in a number of studies that do not relate these data to pesticide exposure or to behaviors that might predict exposure. Quandt et al. (1998, 2001) identified several key beliefs held by farmworkers that might increase behaviors that would promote pesticide exposure. These beliefs include the ideas that pesticides must be felt, seen, tasted, or smelled to be present; the skin blocks absorption and body openings facilitate it; exposure occurs only when a pesticide is wet; susceptibility is individualized; and acute, not low–level chronic, exposure is the primary danger inherent in pesticide exposure. Elmore and Arcury (2001) found similar beliefs among Christmas tree workers. Salazar et al. (2004) found that workers expected to get sick as part of the job. They believed it was all right to work in unsafe conditions if the benefits were high enough. Hunt et al. (1999) found similar beliefs in southern Mexico.

In research with pesticide applicators, Martinez et al. (2004) found that applicators believe, in contrast to farmworkers, that dermal exposure is linked to long-term adverse health consequences, but not to acute illness. The knowledge and beliefs held by applicators reflect their participation in required training (Martinez et al. 2004; Perry et al. 2000). Much of it appears to have been learned by rote with less than optimal understanding of the health consequences of exposure.

Some studies have tried to measure the association of pesticide knowledge and beliefs with pesticide-related behavior. These studies (Arcury et al. 2002; Grieshop et al. 1996; McCauley et al. 2002; Vaughan 1993) show that greater knowledge of pesticide risks increases workers’ sense of control and willingness to practice safety behaviors that should reduce exposure. Among farm operators, the belief that one had previously experienced adverse events of exposure was linked to taking greater precautions when working with pesticides (Lichtenberg et al. 1999).

### Values and folk beliefs

Familism (an orientation to the welfare of one’s immediate and extended family) has been noted as a strong value among Mexican and Central American immigrants (Romero et al. 2004; Sabogal et al. 1987; Salazar et al. 2004). Among adolescent farmworkers, this value is so strong that researchers (e.g., Salazar et al. 2004) have suggested that these workers are likely to neglect themselves (e.g., not adhere to safety practices) in their agricultural work with pesticides. Other authors (e.g., Romero et al. 2004; Sabogal et al. 1987) have suggested that familism should be associated with more positive health outcomes. Thus, of farmworkers who have been exposed to pesticides, those with greater familism may experience lower rates of pesticide-related illness.

Two folk illness concepts that are characteristic of Mexico have been identified among farmworkers. “Susto,” an illness associated with having experienced a fright (Rubel 1984), was reported by a significant number of Mexican farmworkers in Florida who had experienced pesticide exposure (Baer et al. 1993). Arcury et al. (2001) reported that farmworkers expressed reluctance to use cold water for washing in the field and to shower immediately after returning home from work. They attributed this reluctance to a concern (indicative of a belief in humoral medicine) (Rubel 1960; Weller 1983) that their bodies were metaphorically hot from work and that the contact with water that, despite variation in temperature, is metaphorically cold, would result in rheumatism and other adverse health outcomes. These studies suggest that folk beliefs about the causes of illness can promote greater pesticide exposure by undermining protective behaviors such as hand washing and using PPE.

## Summary of the Evidence

While many diverse factors have been proposed to have direct, indirect, or modifying effects on whether or not farmworkers are exposed to pesticides (Table 1; Figure 1), the research connecting characteristics of workers’ environments and behaviors with actual measures of pesticide exposure is meager. Behavioral factors for which the best evidence of a direct relationship with pesticide exposure exists are use of PPE, use of pesticide products in and around the home, and personal hygiene behaviors such as hand washing at work and showering upon returning home from work.

Evidence of environmental factors associated with exposure is lacking for the occupational setting. Aside from clear evidence that job tasks that bring workers into contact with pesticides produce greater exposure, there has been little attempt actually to measure the effect of workplace safety training or the organization of work on exposure. Far more attention has been paid to the effects of the household environment of farmworkers and applicators on the exposure of workers and family members because we have better access to homes than to work sites. With some exceptions, research supports the link between proximity to fields and exposure. While studies use different measures, older houses of poorer quality appear to be linked to exposure. Similarly, different measures of household composition have been used. Most suggest that a greater number of adults and farmworkers in a house leads to greater amounts of pesticide in the dwelling and more pesticide exposure of the residents.

None of the psychosocial or cultural factors proposed as moderators in the association of environment or behavior with exposure has been examined with actual pesticide exposure data. Thus, the role of such factors in farmworker exposure is unknown.

The review of the evidence also highlights the fact that many of the existing studies that identify predictors of pesticide exposure in farmworkers, as well as in nonfarmworkers, have relied on self-reported behaviors rather than on true exposure measures. Among those studies that have included measures of exposure, some have employed environmental samples rather than biological measures. This history suggests that further studies of the association between predictors of exposure and actual biomarkers are warranted.

## Recommendations for Data Collection and for Future Research

The evidence provided by this review, encompassing both factors with demonstrable links to exposure and those plausible but not well studied, indicates that a minimum set of concepts should be included in studies of farmworker pesticide exposure. The exact measures for each concept are not entirely clear because of the dearth of research that has actually sought to measure the association of predictors and exposure outcomes. Therefore, the recommendation is to obtain a broad enough group of measures to test for likely pathways of exposure.

This minimum set differs depending on whether the research focus is limited to occupational pesticide exposure of workers or if the focus includes the paraoccupational and environmental pesticide exposure of adults and children who reside with farmworkers. For the latter, some additional measures are included (e.g., child play areas). Measures are presented from proximal to distal determinants (Table 2). Although this review has included a variety of moderators that are likely to be important in the exposure pathway, there is currently insufficient research to recommend any particular set of such measures.

## Future Research

This review suggests that a productive line of research would be to focus on the role of the organization of work with regard to pesticide exposure. This area of research can help identify aspects of the workplace that can be modified to protect workers from pesticide exposure. It is consistent with the approach of much of occupational safety and health, in that it relies less on changing human behavior directly than on “engineering” changes in work and the workplace environment. While the organization of work is a well developed area of research, it has not had widespread application to farmworker pesticide safety research.

The most obvious dearth of data found in this review is in the area of cultural and psychosocial factors that may moderate the effect of household and workplace environments on safety behaviors. Although such factors are clearly not direct influences on exposure, they condition the extent to which behavior or environmental change to protect workers and their families will be accepted, and they are, therefore, necessary components of behavioral interventions. It is premature to list specific data to be collected because such factors do not lend themselves to measurement through simple questions.

## Figures and Tables

**Table 1:** Review of literature on predictors of pesticide exposure among migrant and seasonal farmworkers.

	Relationship to pesticide exposure				
Characteristic	Rating^a^	Reference	Population	Exposure measurement	Findings
Workplace behaviors					
Availability and use of personal protective equipment	1	Fenske et al. 1990	12 farmworkers	Dermal exposure to lindane	Demonstrated penetration of lindane through workshirt and pants. Recommended adding coveralls and gauntlet-type gloves
	1	Gomes et al. 1999	532 farmworkers in United Arab Emirates	Blood sample: Acetylcholinesterase (AChE) activity	Higher AChE was associated with changing work clothes and use of work coveralls, gloves, and face scarf
	1	Lander et al. 1991	100 greenhouse workers and 43 fruit growers; 113 slaughtermen served as controls	Blood sample: AChE activity	Wearing gloves was protective of AChE activity in greenhouse workers
	1	Ohayo-Mitoko et al. 1999	539 agricultural workers in 4 areas of Kenya	Blood sample: AChE activity	Use of coverall resulted in less AChE inhibition than not wearing coverall or just wearing boots
	1	Spencer et al. 1995	28 peach harvesters, California	Dislodgeable foliar residue of azinphos-methyl(AM) pesticides measured on skin and clothing	More pesticides were found on outer of two shirts, indicating the protective effect of clothing from dislodgeable residues
	1	Hernández-Valero et al. 2001	26 Mexican American migrant farmworkers in Baytown, Texas	Blood samples: 21 organochlorine pesticides(OCPs)	Wearing gloves and hats resulted in less OCP exposure in farmworkers than wearing only hats
	2	Arbuckle et al. 2002	126 pesticide applicators in Ontario	Urine samples: Phenoxy-herbicides 2,4-dichlorophenoxyacetic acid (2,4-D) or 4-chloro-2-methylphenoxyacetic acid (MCPA)	Reduced pesticide in urine following application was associated with use of rubber gloves for mixing/loading, and wearing rubber boots for cleanup
	2	Fenske et al. 2002a	6 pesticide applicators in central Florida citrus groves	Exposure to organophosphorus (OP) insecticide ethion during airblast application by fluorescent tracer deposition on skin surfaces beneath garments, video imaging analysis instrument (VITAE system), and alpha-cellulose patches placed outside and beneath the garments	Among applicators, compared dermal exposure to pesticides for cotton work shirts/pants, woven coveralls, nonwoven garments. All garments allowed fabric penetration. Exposure was highest with nonwoven garments, mostly because of large sleeve and neck openings
	2	Nigg et al. 1993	3 greenhouse pesticide applicators in Florida	Pads placed inside and outside three types of protective coveralls measured exposure to chlorpyrifos, fluvalinate, and ethazol	Less penetration of synthetic disposable coverall than of reusable treated twill coverall
Field sanitation	1	Curwin et al. 2003	12 Hispanic male tobacco harvesters near Kinston, North Carolina	Handwipes: acephate residues	Farmworkers removed 96% of acephate on hands by washing
Household behaviors					
Residental pesticide use	1	Arcury et al. 2005	9 Latino farmworker family households in western North Carolina and Virginia	Urine samples: OP metabolites	Residential pesticide use was associated with higher levels of OP metabolites in samples from children and adults living in farmworker dwellings
	1	Bradman et al. 1997	5 farmworker and 6 nonfarmworker dwellings in California’s Central Valley	House dust and handwipe sample: 33 pesticides	Residential application of agricultural and residential pesticides was related to presence of pesticides in dust samples
	1	McCauley et al. 2001	96 farmworker homes and 24 grower homes in two agricultural communities in Oregon	House dust samples: residues of major OPs used in area crops	Found no relationship between pesticides in wipe samples and “family use of pest control products”
	1	Quandt et al. 2002, 2004	41 farmworker family homes in North Carolina and Virginia	Wipe samples from floor, toys, and children’s hands: 8 locally reported agricultural pesticides and 13 pesticides commonly found in U.S. houses	Found a greater number and weight of residential pesticides than agricultural pesticides in dust samples collected in farmworker dwellings
	2	Fenske et al 2002b	12 farmworker homes in Central Washington State; 14 nonagricultural reference homes	House dust samples and children’s urine samples: 2 diethyl OP pesticides—chlorpyrifos and parathion	OP pesticide use in garden was associated with increased metabolite concentrations in children’s urine
	2	Lewis et al. 2001	Single household	Samples of indoor air; vacuumable carpet dust; carpet dislodgeable residues; deposits on bare floors, table tops, and dinnerware; surrogate food; and residues on children’s hands and toys: diazinon and chlorpyrifos	Demonstrated that indoor and outdoor residential pesticide application resulted in pesticides on surfaces in homes accessible to human contact
	2	Lu et al. 2001	110 children, ages 2–5 years, from 96 households in the Seattle metropolitan area	Urine samples: 6 dialkylphosphate (DAP) compounds	Children’s OP pesticide concentrations were higher if parents reported garden pesticide use but were not based on indoor residential pesticide use
	2	McCauley et al. 2003	24 agricultural families in northwestern US	House dust samples: OP pesticides	Pesticide use in the home was not related to levels of total OP residues
	2	Morgan et al. 2001	Single family dwelling in Chatham County, North Carolina	Soil, turf, and carpet samples; 24-hr air samples; handwipes; and samples taken from dog fur and paws	Children and adults were exposed to pesticides that were applied to yards and then were transferred into the house by pets (dogs), adults, and children
	2	Nishioka et al. 2001	11 occupied and 2 unoccupied homes	Indoor air samples; surface wipes from floors, table tops, and window sills; and floor dust samples before and after lawn application of the herbicide 2,4-D	Children and adults were exposed to pesticides that were applied to yards and then were transferred into the house by pets (dogs) and adults
Cleaning	1	Arcury et al. 2005	9 Latino farmworker family households in western North Carolina and Virginia	Urine samples: OP metabolites	Living in a dwelling that is easier to clean and that has a vacuum cleaner was associated with lower levels of OP metabolites among children and adults
	1	Bradman et al. 1997	5 farmworker and 6 nonfarmworkers dwellings in California’s Central Valley	House dust and handwipe samples: 33 pesticides	Frequency and type of cleaning (mopping, vacuuming) was related to presence of pesticides in dust samples
Laundry	1	Arcury et al. 2005	9 Latino farmworker family households in western North Carolina and Virginia	Urine samples: OP metabolites	Higher levels of OP metabolites for adults and children were associated with improper handling of laundry, including storage of work clothes in house and laundering of work clothes with family clothes
Delay changing clothes and bathing	1	Arcury et al. 2005	9 Latino farmworker family households in western North Carolina and Virginia	Urine samples: OP metabolites	Higher levels of OP metabolites for adults and children were associated with farmworkers who delay changing from work clothes and bathing
	2	McCauley et al. 2003	24 agricultural families in northwestern US	House dust samples: OP pesticides	Level of total OPs and of azinphos-methyl was higher in homes where workers waited > 2 hr before changing out of work clothes
Household pets	2	Lu et al. 2001	110 children, ages 2–5 years, from 96 households in the Seattle metropolitan area	Spot urine samples: six dialkylphosphate (DAP) compounds	OP pesticide concentrations in children were not different based on reported pet treatment
	2	McCauley et al. 2003	24 agricultural families in northwestern US	House dust samples: OP pesticides	Total number of pets in the home was not related to levels of total OP residues
	2	Morgan et al. 2001	Single-family dwelling in Chatham County, North Carolina	Soil, turf, and carpet samples; 24-hr air samples; handwipes; and samples taken from dog fur and paws: pesticides	Pet dog was a vehicle for the transfer of pesticide residues from lawn to house
	2	Nishioka et al. 2001	11 occupied and 2 unoccupied homes	Indoor air samples; surface wipes from floors, table tops, and window sills; and floor dust samples: before and after lawn application of herbicide 2,4-D	Pet dog was a vehicle for the transfer of pesticide residues from lawn to house
Child activity patterns	2	Morgan et al. 2001	Single–family dwelling in Chatham County, North Carolina	Soil, turf, and carpet samples; 24-hr air samples; handwipes, and samples taken from dog fur and paws: pesticides	Children were a vehicle for the transfer of pesticide residues from lawn to house
	2	Mandel et al. 2005	95 farm families (grower, spouse, and child) in Minnesota and South Carolina	24-hr urine samples: 2,4-D; glyphosphate; and metabolite of chlorpyrifos	Children’s urine pesticide concentrations were lower than those of growers, but higher than those of growers’ spouses, thus reflecting children’s activity patterns
Diet	2	Curl et al. 2003	39 preschool age children (18 children with organic diets and 21 children with conventional diets) in Seattle, Washington	24-hr urine samples: 5 OP pesticide metabolites	Urine of children who ate an organic diet contained significantly lower levels of OP metabolites than urine of those who ate a conventional diet
	2	Stehr-Green et al. 1988	85 rural-dwelling persons	Blood samples: 11 pesticide residues and metabolites	In “rural-dwelling persons,” consumption of home-produced eggs and root vegetables was associated with increased serum concentrations of pesticides
Transportation	1	Curl et al. 2002	218 farmworker households in Washington State	House and vehicle dust samples: 6 pesticides Urine samples: 5-dialkylphosphate (DAP) metabolites	Found pesticides in dust samples collected in farmworker vehicles
	1	Thompson et al. 2003	571 farmworkers in the Lower Yakima Valley in Washington State	Urine samples of farmworkers and children, house and vehicle dust samples: pesticides	Found pesticides in dust samples collected in farmworker vehcles
Workplace environment					
Task variety	1	Hernandez-Valero et al. 2001	26 Mexican American migrant farmworkers in Baytown, Texas	Blood samples measured 21 organochlorine pesticides (OCPs)	Number of tasks that brought farmworkers into contact with pesticides was associated with elevated serum levels of mirex, DDT, and *trans*-nonachlor
Job design	1	Coronado et al. 2004	213 farmworkers in 24 communities and labor camps in eastern Washington State	Urine samples: OP metabolites; House and vehicle dust samples: OP pesticides	Workers performing tasks not regulated by WPS (e.g., thinning) were more likely to have detectable levels of azinphos-methyl in house and vehicle dust
Household environment: dwelling characteristics					
Dwelling (location relative to exposure sources)	1	McCauley et al. 2001	96 farmworker homes and 24 grower homes in two agricultural communities in Oregon	Home dust samples: OP residues	Found that azinphos-methyl concentration decreased with increased distance from fields
	1	Curl et al. 2002	218 farmworker households in Washington State	House and vehicle dust samples: 6 pesticides Urine samples: 5 OP metabolites	Strong correlation between pesticides in cars and in house dust. Weaker correlation between house dust and child urine. No association between distance to fields and child’s urine, thus suggesting that behavior, not proximity to fields, was responsible for exposure
	1	Quandt et al. 2002, 2004	41 farmworker family residences in North Carolina and Virginia	Wipe samples from floor, toys, and children’s hands: 8 eight locally reported agricultural pesticides and 13 pesticides commonly found in U.S. houses	Proximity to agricultural fields was related to the number of agricultural pesticides detected in dust samples collected in dwellings
	2	Fenske et al. 2002b	12 farmworker homes in Central Washington State and 14 nonagricultural reference homes	House dust samples and children’s urine samples: chlorpyrifos and parathion	Homes in close proximity (200 ft/60 m) to pesticide-treated farmland had higher chlorpyrifos and parathion house dust concentrations than did homes farther away, but this effect was not reflected in the urinary metabolite data
	2	Loewenherz et al. 1997	88 children under 6 years in 48 pesticide applicator and 14 reference families	Urine samples: OP metabolites	Higher DMTP levels were found in applicator children living < 200 ft from an orchard than in nonproximal applicator children
	2	Lu et al. 2000	109 children, 9 months to 6 years, in an agricultural community in central Washington State	Urine and hand wipe samples: OP pesticides House dust samples and wipe samples: OP pesticides	Higher levels of pesticides were found in dust samples from dwellings closer to orchards
Dwelling type	1	McCauley et al. 2001	96 farmworker homes and 24 grower homes in two agricultural communities in Oregon	Home dust samples: residues of major OPs used in area crops	Housing type (labor camp, trailer, apartment) was not related to pesticide residues
Dwelling tenure	1	Arcury et al. 2005	9 Latino farmworker family households in western North Carolina and Virginia	Urine samples: OP metabolites	Renting rather than owning was associated with higher levels of OP metabolites found in samples from persons living in farmworker dwellings
Housing quality/state of repair	1	Bradman et al. 1997	5 farmworker and 6 nonfarmworker dwellings in California’s Central Valley	House dust and handwipe sample: 33 pesticides	Dwelling age is related to presence of pesticides in dust samples
	1	Quandt et al. 2002, 2004	41 farmworker family residences in North Carolina and Virginia	Wipe samples from floor, toys, and children’s hands: 8 locally reported agricultural pesticides and 13 pesticides commonly found in U.S. houses	More residential pesticides were found in dust samples collected in dwellings judged to be difficult to clean
Household environment: household characteristics					
Total household size (total number of residents)	1	Arcury et al. 2005	9 Latino farmworker family households in western North Carolina and Virginia	Urine samples: OP metabolites	Larger household size was associated with higher levels of OP metabolites for adults and children
	1	McCauley et al. 2001	96 farmworker homes and 24 grower homes in two agricultural communities in Oregon	Home dust samples: OP residues	More persons in household was related to greater azinphos-methyl in dust
	2	McCauley et al. 2003	24 agricultural families in northwestern United States	House dust samples: OP pesticides	Weak, nonsignificant correlation was found between number of household residents and levels of total OP residues. Number of adults in household
	1	Arcury et al. 2005	9 Latino farmworker family households in western North Carolina and Virginia	Urine samples: OP metabolites	More adults in the household was associated with higher levels of OP metabolites for adults and children
Number of farmworkers in household	1	McCauley et al. 2001	96 farmworker homes and 24 grower homes in two agricultural communities in Oregon	Home dust samples: OP residues	More farmworkers in household was related to greater azinphos-methyl in dust
	1	Bradman et al. 1997	5 farmworker and 6 nonfarmworkers dwellings in California’s Central Valley	House dust and handwipe sample: 33 pesticides	Higher amounts of pesticides in dust in farm worker than nonfarmworker homes. Pesticides found on hands of children in farmworker, but not nonfarmworker homes, suggest take home pesticides
	2	Lu et al. 2000	109 children, 9 months to 6 years of age, in an agricultural community in central Washington State	Urine and hand wipe samples: OP pesticides. House dust samples and wipe samples from various surfaces: OP pesticides	Households with agricultural workers had higher levels of OP pesticides in dust wipe samples and on children’s hands, and higher levels of metabolites in children’s urine samples, than reference homes
	2	Simcox et al. 1995	26 farming, 22 farmworker, and 11 nonfarming residences in eastern Washington State	House dust and soil samples: 4 OP insecticides	OP pesticide residues were found more often in homes of agricultural workers than in reference homes
Household composition	1	Arcury et al. 2005	9 Latino farmworker family households in western North Carolina and Virginia	Urine samples: OP metabolites	Higher levels of OP metabolites for adults and children were associated with nonnuclear family household composition
	1	Quandt et al. 2004	41 farmworker family residences in North Carolina and Virginia	Wipe samples from floor, toys, and children’s hands: 8 locally reported agricultural pesticides and 13 pesticides commonly found in U.S. houses	Nonnuclear family household composition was weakly associated with agricultural but not with residential pesticides
Household density or crowding	1	McCauley et al. 2001	96 farmworker homes and 24 grower homes in two agricultural communities in Oregon	Home dust samples: OP residues	Found no relationship between pesticides and area of home
	2	McCauley et al. 2003	24 agricultural families in northwestern United States	House dust samples: OP residues	Weak correlation was found between total area of home and levels of total OPs residues
Community environment					
Overall level of agricultural pesticide use	1–2	Fenske et al. 2000	109 children in agricultural community in eastern Washington State (91 had parents working in agriculture)	Urine samples: OP metabolites	Most children living in an agricultural region during the spray season had measureable dialkyphosphates, and a substantial fraction had doses > reference values for azinphos-methyl
	2	Koch et al. 2002	44 children living in an agricultural community in central Washington State	Urine samples: dialkylphosphate (DAP) metabolites	DAP metabolites were elevated when OP pesticides were sprayed in the region. No differences were found to be related to parental occupation or residential proximity to fields
	2	Lee et al. 2002	California communities	Ambient air sampling of multiple classes of airborne pesticides	Exposure estimates ≥ risk of noncancer health effects reference values occurred for 50% of exposed population for several pesticides
Historical agricultural pesticide use	2	Wolz et al. 2003	58 homes in agricultural community in Washington State	Soil and house dust samples: lead arsenate	Dwellings near land used for orchard production during 1905–1947 had significantly higher soil and household lead, and also higher soil arsenic than other homes
	2	Miersma et al. 2003	Elementary school yards in 8 cities near the Texas–Mexico border	Soil samples: OCPs	Attributed OCPs found in school yards to historical agricultural activity

a 1 = Association with pesticide exposure was demonstrated in farmworkers. 2 = Association with pesticide exposure was demonstrated in nonfarmworker samples.

**Table 2:** Recommended measures of predictors of pesticide exposure among migrant and seasonal farmworkers.

Workplace behaviors	Wear clean clothes to work (frequency)
	Wash hands at work (frequency)
	Use of personal protective equipment (type, frequency)
Household behaviors	Residential use of pesticides (type, frequency), including pet products
	Wear work clothes into dwelling
	Wear work shoes into dwelling
	Time to changing from work clothes after work
	Time to bathing after work
	Contact with others before changing clothes after work
	Contact with others before bathing after work
	Storage of soiled work clothes
	Laundry method (machine, hand)
	Separation of work and family clothes in laundry
	Child play areas (inside, outside)
Work environment	Safety training (contents, quality)
	Work task (fieldwork, mix and load, apply)
	Access to hygiene facilities
	Availability of personal protective equipment
	Ability to communicate with supervisor
Residential environment	Location relative to pesticide application
	Housing structure type
	Housing overall repair
	Housing size (area, rooms)
	Bathing facilities per resident
	Laundry facilities per resident
	Total number residents
	Total number of farmworkers
	Crowding; adult/room; workers/room; workers/sleeping room
Community environment	Agricultural acreage
	Volume pesticides applied/year
